# Breastfeeding Attitudes Among Female Students in Syria and Hungary

**DOI:** 10.3390/nu17132121

**Published:** 2025-06-26

**Authors:** Manar Al Kamsheh, Krisztina Antónia Bornemissza, Alexandra Zimonyi-Bakó, Helga Judit Feith

**Affiliations:** 1 Health Sciences Division, Doctoral College, Semmelweis University, 1085 Budapest, Hungary; manarkamsheh@gmail.com; 2 Faculty of Humanities and Social Sciences, Pázmány Péter Catholic University, 1088 Budapest, Hungary; bornemissza.krisztina.job@gmail.com; 3 Institute of Languages for Specific Purposes, Semmelweis University, 1091 Budapest, Hungary; bako.alexandra@semmelweis.hu; 4 Department of Social Sciences, Faculty of Health Sciences, Semmelweis University, 1085 Budapest, Hungary

**Keywords:** breastfeeding, formula-feeding, Syria, Hungary, attitude, IIFAS, infant, university students, female

## Abstract

**Background:** Breastfeeding is the ideal source of nutrition for babies. Despite its benefits, breastfeeding practices and attitudes vary across cultures, influenced by societal norms, education, and personal experiences. This article shows the attitude differences among female students in Syria and Hungary and how sociocultural aspects impact their attitude towards breastfeeding. **Methods:** The questionnaire was a part of a multi-section questionnaire presented to 317 Syrian students and 303 Hungarian students. It assessed students’ attitudes towards breastfeeding through the Iowa Infant Feeding Attitude Scale (IIFAS). In addition to cross-tabulations, an exploratory data categorisation method, i.e., cluster analysis, was used in analysing the data. **Results:** Participants in both countries demonstrated strong agreement with statements highlighting the emotional and nutritional advantages of breastfeeding. In total, 67.2% of the participants disagreed with the idea that formula feeding is more convenient; similarly, 66.3% of them disagreed that breastfeeding causes fathers to feel emotionally excluded. Attitudes toward breastfeeding in public were more divided, reflecting the sensitivity of the topic and varying degrees of acceptance, with 48.7% of respondents disagreeing. Multivariate analysis demonstrated that nationality or age were significant predictors of belonging to various attitude clusters (SBM, SFF, FT): Syrian respondents and younger participants aged 21–30 years were more likely to belong to the Supporters of Breast Milk (SBM) cluster. In addition, paternal education level and urban residence also influenced feeding attitudes. **Conclusions:** This study shows the differences in attitude among Syrian and Hungarian female students, which is rooted in cultural diversity and its effect on individuals’ decisions.

## 1. Introduction

Breastfeeding is well recognised as the best source of nutrition for infants [1,2,3], providing both mother and infant with numerous health benefits and long-term advantages [1,4,5,6]. Human breast milk is specifically adapted to the infants’ biological requirements and is the optimal source of sustenance for infants [2]. Breastfeeding protects against diarrhoea and common childhood illnesses such as pneumonia, can have longer-term advantages, like lowering the risk of childhood and adolescent overweight and obesity [1,7,8,9], and provides necessary support for the developing immune system [10,11]. Exclusive breastfeeding is related to low levels of morbidity [9,12] and fewer hospitalisations [13]. Centres for Disease Control and Prevention in the United States (CDC) consider breastfeeding the optimal form of nutrition for infants [14].

Similar to its protective effects against various diseases in infants, it has several benefits for mothers, including reducing the incidence of some diseases. Women who breastfeed have a lower risk of developing breast cancer, type 2 diabetes, ovarian cancer, heart disease, osteoporosis, and postpartum depression [4,7,15,16,17].

Additional benefits of breastfeeding include economic advantages. Breast milk is less expensive than formula; due to its natural secretion from the mother’s body, breast milk is free and always available. Although some mothers pump breast milk using a special pump, store it in the freezer in specific storage bags and then warm it to introduce it to their baby, in most cases, there is no need to pay for, prepare or store breastmilk as opposed to formula. Formula feeding generates extra costs: formula, bottles, sterilising equipment, and, in exceptional cases, special types of formula for allergic babies or those with specific dietary needs. In fact, most often, through breastfeeding, families avoid these expenses, and therefore, it is more economical and accessible [18,19,20].

Nonetheless, infant formula is still a healthy alternative for mothers who cannot or decide not to breastfeed. While it provides babies with the nutrients they need to grow and thrive, its benefits are still fewer than those of breastmilk [21]; therefore, manufacturers have proposed a diverse range of new ingredients in an effort to develop formulas that mimic the perceived and potential advantages of human milk [22].

Differences in attitudes toward breastfeeding can be attributed to cultural norms [23,24,25], societal expectations, social networks [26], personal experiences [27,28], and exposure to breastfeeding education [29]. In certain societies, breastfeeding is seen as a duty for all mothers [30,31,32], while in some others, formula feeding is accepted as an alternative [33,34]. Additionally, media representation, healthcare recommendations, and prior exposure to breastfeeding within one’s social environment play a significant role in shaping attitudes [25,35].

Some countries like Korea and Albania have high exclusive breastfeeding rates, with almost 71% and 62%, respectively, according to UNICEF data, while in the United States, for example, the rate of exclusive breastfeeding until five months is only of 26% [36].

The prevalence of breastfeeding in Hungary is 53.9%, while that of exclusive breastfeeding is 25.1% [37]. These numbers are higher than in the WHO European Region, which has some of the lowest rates of exclusive breastfeeding, with just 13% of infants exclusively breastfed for the first 6 months [38].

Education can impact students’ perspectives in terms of breastfeeding knowledge, resulting in more evidence-based views [29], while those with limited information may rely on societal norms or personal opinions [39]. These variations highlight the interplay between cultural, social, and educational factors in shaping attitudes toward infant feeding.

Although studies documenting breastfeeding attitudes have been reported previously in a range of settings [40,41,42,43,44], this is the first time, to our knowledge, that the comparison in attitudes between Syrian and Hungarian female university students has been assessed using the Iowa Infant Feeding Attitude Scale (IIFAS).

This study aims to understand how attitudes can influence future mothers’ decisions. Focusing on university students who represent future parents helps to understand the young generation’s attitude towards breastfeeding, providing insights into how cultural and educational factors impact breastfeeding beliefs, which may be helpful for international public health initiatives.

This study fills a gap by comparing two distinct populations, Syrian and Hungarian university students, who differ in cultural norms, healthcare exposure, and social expectations [25]. Exploring these differences can also inform future interventions that address misconceptions, promote breastfeeding awareness, and support women’s choices in distinct cultural contexts.

## 2. Materials and Methods

### 2.1. Participants

The survey was conducted in Syria and Hungary, in two phases. The first phase occurred at Damascus University (Faculty of Pharmacy and the Faculty of Arts and Humanities) in October and November 2022, with a sample of 317 female students. The second phase was conducted in Budapest, Hungary, during April and May 2023, involving 303 Semmelweis University (Faculty of Pharmacy) and Eötvös Loránd University (Faculty of Art) students. Due to the research design, the inclusion of the respondents in the survey was not carried out in the context of a research topic specific course; however, it was important to survey (1) full-time students among (2) health and non-health orientations, (3) BSc, MSc, and PhD programmes, and (4) different year groups. Responses were collected in a classroom setting, but on an anonymous and voluntary basis in accordance with research ethics permissions in Syria and Hungary, after signing appropriate information and consent forms (Table 1). To ensure linguistic accuracy, the questionnaire was administered exclusively in paper format in the native languages of each country: Arabic and Hungarian. Independent professional translators translated the survey from English to Arabic and Hungarian using the back-translation method.

For the Syrian data collection, the survey was sent to 389 potential participants, of whom 317 completed and sent in valid responses, resulting in a response rate of 81.5%. The survey was organised separately in Hungary. There, the questionnaire was sent to 350 persons, of whom 303 respondents returned valid responses, which resulted in a response rate of 86.5%.

### 2.2. Study Design

This research is part of an extensive survey using a multi-section questionnaire with three modules [25]. The present study investigates the Iowa Infant Feeding Attitude Scale (IIFAS) (the Iowa Infant Feeding Attitude Scale (IIFAS) can be found in Table 3), which is a widely used tool designed to assess attitudes about infant-feeding methods, including breastfeeding and formula feeding, developed by De La Mora and Russell in 1999 [45]. The scale evaluates individuals’ beliefs and preferences regarding infant-feeding practices. The participants were required to read the statements and choose the most appropriate response from a 5-point Likert scale, ranging from “strongly disagree” to “strongly agree, with the “not sure” option provided.

The IIFAS statements can be grouped by four thematic criteria: participants’ attitudes toward perceived health and nutritional values, convenience and practical aspects, bonding and emotional connection, and social and risk perception. These four criteria were identified among the participants’ attitudes and reported in detail in the Discussion section.

### 2.3. Socio-Demographic Characteristics of the Samples

This study utilised a questionnaire to collect data on the participants’ socio-demographic characteristics, including nationality, gender, birth year, parental education levels, marital status, permanent residency, and wealth index. Additionally, the participants’ current level of education was recorded (Table 2).

Comparing the Hungarian and the Syrian samples revealed significant differences in several socio-economic dimensions. In terms of educational level (Sig.: 0.000; Cramer’s V: 0.264), a larger percentage of Syrians (79.5%) had a BSc degree than Hungarians (57.1%), while Hungarians had a higher percentage of MSc degrees (37.0% vs. 14.2%). By place of residence (Sig.: 0.000; Cramer’s V: 0.208), a significantly higher percentage of Syrian respondents lived in an urban neighbourhood (82.3%) than Hungarians (63.9%). Marital status presented a marked difference (Sig.: 0.000; Cramer’s V: 0.596): while almost all Hungarian respondents were unmarried (99.7%), more than half of the Syrian respondents (53.6%) were married. The wealth indicator showed the greatest imbalance (Sig.: 0.000; Cramer’s V: 0.687): almost all Hungarians (97.4%) said they had “enough” income, while two-thirds of Syrians (66.8%) said they had “less than enough” for their necessities. Finally, the distribution of age showed (Sig.: 0.000; Cramer’s V: 0.246) that most respondents in both groups were in the younger age category (21–25 years).

The samples from the two countries were nearly balanced regarding nationality, with 51.0% Syrian and 49.0% Hungarian respondents. All participants were female, with the majority, 68.6%, enrolled in bachelor’s programs, 25.3% pursuing master’s degrees, and a smaller proportion, 6.1%, engaged in PhD studies.

### 2.4. Measurements

Statistical procedures were completed at a significance level of 5%. Descriptive statistics were performed for demographic variables, scale scores, and the responses to all statements within each scale.

IBM SPSS Statistics Data Editor software (SPSS 25.0) was used for data analysis. Cross-tabulation analyses were carried out primarily to obtain a clearer picture of the relationships. Cluster analysis was used to create clusters based on the responses, and starting from the cluster groups, we observed the demographic characteristics of those with different attitudes and the differences between the groups, including differences between nationalities. Cluster analysis was carried out because it is an excellent way of identifying common attitudinal patterns between nationalities, or even those that are present in different ways within nationalities. It was used as a kind of complementary analysis to compare nationality groups.

Although no prior sample size calculation was performed, a post hoc power analysis was conducted based on the effect size (Cramer’s V = 0.502) observed in the comparison of nationality and cluster membership. With the available sample size (N = 620), the statistical power exceeded 0.99 to detect medium–large effects (α = 0.05), indicating that the sample was fully adequate to identify significant group differences.

## 3. Results

The findings of this study regarding the comparison between Hungarian and Syrian students present the distribution of responses to the IIFAS, highlighting their attitudes toward breastfeeding (Table 3).

### 3.1. Agreement and Disagreement

The most vigorous agreement was in the statement discussing whether breastfeeding boosts the connection between mother and infant; it can be observed that 95.8% of participants agree with this statement (Table 3). Similarly, 87.9% agreed that breast milk is the ideal food for babies. Likewise, slightly fewer, but still 62.1% of participants, agreed that breastfed babies are healthier than formula-fed babies.

The disagreements among students were shown in many statements; 67.2% of participants disagreed that the formula is more suitable or comfortable than breastfeeding, while 66.3% of participants showed disagreement that fathers feel left out if a mother breastfeeds. Regarding the statement related to breastfeeding in public places, the participants showed 48.7% disagreement with not breastfeeding publicly.

Many differences emerged in participants’ responses, one of is which related to the statement that breast milk is more easily digested than formula (Sig.: 0.000; Cramer’s V: 0.519). While 83.0% of Syrian participants agreed with this statement, only 34.8% of Hungarian participants shared this agreement. Similarly, 77.6% of Syrian participants agreed with the statement that mothers who formula-feed miss one of the great joys of motherhood. In comparison, only 30.7% of Hungarian students agreed with this sentiment (Sig.: 0.000; Cramer’s V: 0.586).

Hungarian students were notably more uncertain about the statement “*Breast milk is lacking in iron*”: 71.5% of Hungarian participants were unsure about this claim, while 53.3% of Syrian participants opposed it (Sig.: 0.000; Cramer’s V: 0.503). Along similar lines, for the two statements about overfeeding, which have opposite meanings, the percentages of participants who selected “not sure” were notably high and comparable across groups. Regarding the statement “*Formula-fed babies are more likely to be overfed than are breastfed babies*”, 38.9% of participants were unsure (Sig.: 0.000; Cramer’s V: 0.203), while for “*Breastfed babies are more likely to be overfed than formula-fed babies*” 38.1% expressed uncertainty (Sig.: 0.000; Cramer’s V: 0.226) (Table 3).

### 3.2. Cluster Analysis

**Figure 1:**
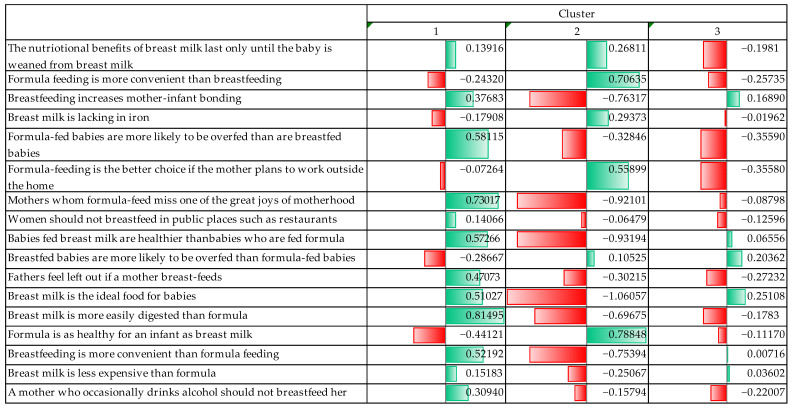
Clusters of attitudes toward breastfeeding and formula feeding. (Red: "Negative (minus) values"; Green: "Positive (plus) values).

Based on the attitudes towards breastfeeding and formula feeding, the cluster analysis detected three well-distinct clusters. In the following, the process of cluster formation is firstly described and a description of the clusters that emerged is provided (Figure 1).

In the process of clustering, it was observed that the groups were well separated, and the differences between the clusters became explicitly clear. The maximum distance between cluster centres was 10.257, meaning the respondents’ opinions were explicitly divergent.

The significance level for all variables used for cluster analysis was below 0.05, indicating that the differences between clusters were statistically significant. It could be seen during the process that three variables were explicitly determinant and discriminating factors between the clusters. The three variables were positive statements about breastfeeding: “*Mothers who formula feed miss one of the great joys of motherhood*” (F = 212.750); “*Breast milk is the ideal food for babies*” (F = 214.941); “*Breast milk is more easily digested than formula*” (F = 209.261).

The three clusters had distinct characteristics, and based on these characteristics, each cluster was named, the names of which are detailed below.

Our first cluster was called “***Supporters of breast milk***” (***SBM***). Respondents who belong to this cluster explicitly believed in the benefits of breastfeeding and breast milk. They thought breast milk is ideal for babies. In relation to formula feeding, they rejected the idea that formula is as healthy as breast milk and preferred to see the disadvantages; for example, they believed that formula-fed babies are more likely to be overfed. At the same time, cluster members considered breast milk to be more digestible for babies and breastfeeding a more positive experience for mothers. They did not seem to believe that formula feeding is more convenient, nor did they think of breastfeeding in public as a problematic situation. Furthermore, they did not believe that formula feeding is a better option for working mothers.

The second group was called “***Supporters of formula feeding***” (**SFF**). They were those who explicitly accepted the benefits of formula feeding and emphasised the convenience and practicality of formula, especially for mothers returning to work. They did not believe that breast milk makes babies healthier than formula because they believed that the two feeding methods are equally healthy and that by not breastfeeding, a mother is not missing out on an important experience. Even without breastfeeding, they believed that a mother can fully connect with her child and raise it.

The last cluster was called the “***Flexible Thinkers***” (FTs). Respondents in this cluster acknowledged that there are advantages to breast milk, but at the same time, they did not reject formula. They believed that both methods have their advantages and disadvantages. They acknowledged that breast milk is a more natural and healthy food for the baby but rejected extreme pro-breast-milk views. However, they also disagreed with the more extreme pro-formula opinions. Additionally, they did not believe that fathers are missing the experience of raising a child because of breastfeeding. They even favoured breastfeeding but believed that in certain situations it is more practical to use formulas.

Thus, it can be said that in the cluster analysis, the three attitude groups were explicitly distinct on different issues related to breast milk and infant formula. SFF strongly believed in the benefits of breast milk and rejected formulas. SFF believed that formulas are more convenient and healthier. Those with a flexible approach took a balanced view, accepting both methods in certain circumstances.

### 3.3. Sociodemographic Characteristics of Groups Defined by Attitudinal Measurement

After establishing clusters, it is important to examine whether the three groups are separated along sociodemographic lines.

In terms of nationality, two-thirds of Syrian respondents were in the SBM group (61.1%), while only one-tenth of Hungarians thought this way (12.7%). However, the proportion of Hungarians in favour of infant formula feeding was much higher (39.1%), while a tenth of Syrians shared their attitude (13.3%). The proportion of Hungarians thinking flexibly was 48.2%, while it represented only a quarter (25.6%) of Syrian respondents (Table 4).

The relationship between nationality and feeding preferences was significant and moderately strong; so, nationality was a significant factor in determining cluster membership in relation to feeding preferences. Syrian participants were more in favour of breastfeeding, while Hungarians were more flexible or even in favour of formula feeding (Table 4, Sig.: 0.000; Cramer’s V: 0.502).

The proportion of SBM decreased with increasing educational attainment. It was highest among those with a BSc (43.0%), while only a quarter of those with a higher degree fell into this cluster (MSc: 28.1%, PhD: 29.7%). The distribution of the SFF group was relatively even, but the proportion was moderately increased among those with a higher degree (BSc: 24.7%, MSc: 26.7%, PhD: 27.0%). The proportion of FTs was highest among MSc and PhD graduates (MSc: 45.2%, PhD: 43.3%), while a third of those with a bachelor’s degree belonged to this group (32.3%) (Table 5).

Thus, those with MSc and PhD degrees were more likely to be FTs, while those with lower degrees (BSc) were more likely to be in favour of breastfeeding.

Although the relationship between educational attainment and clustering was statistically significant, the level of education had only a moderate influence on feeding attitudes (Table 5, Sig.: 0.012; Cramer’s V: 0.104).

There was also a significant relationship between parental education and respondents’ cluster group membership, but this relationship was very weak (Table 6, Sig.: 0.023; Cramer’s V: 0.113).

A higher proportion (40.5%) of children of fathers who did not have a university degree tended to belong to the SBM group, while a third (35.9%) of children of fathers who had a university degree belonged to this attitude group. Similar proportions were found in the FT group. Among children of fathers with lower education, a higher proportion (38.5%) appeared in this group than among children of fathers with university education (33.2%). The reverse was observed for the proportions of those who were SFF. Children of fathers with a high level of education were more likely to be in the SBM group (30.9%), and children of fathers with a lower level of education were less likely (21.0%) (Table 6).

The following comparison shows that the majority of married respondents (64.1%) were in the SBM group, while the proportion of unmarried respondents was significantly lower in this group. The SFF group was dominated by unmarried respondents (30.6%), while only one-tenth of married respondents belonged to this cluster (12.4%). The FT group included 41.2% of unmarried respondents, while only one quarter of married respondents (23.5%) had similar attitudes. Married people were much more likely to support breastfeeding, while unmarried people were more likely to support formula feeding and to be flexible (Table 7).

Marital status was statistically significant for the feeding preference groups, and a moderately strong effect could be seen (Table 7, Sig.: 0.001; Cramer’s V: 0.336).

Income was also significantly related to the attitudes that made up the clusters, and the association was moderately strong (Table 8, Sig.: 0.001; Cramer’s V: 0.278). The low-income group had the highest proportion of SBM (63.6%), while the lowest proportion was of those in the SFF group (12.1%). Among those with sufficient income, the proportions were more interesting. Both the proportions of SFF (32.9%) and FTs (42.8%) were above 30%; so, they had a higher proportion than SBM. FTs tended to dominate among those with the highest incomes (50.0%), and SFF accounted for 33.3%. Those with lower incomes were more likely to support breastfeeding, while those with higher incomes were more inclined towards formula feeding or a flexible approach (Table 8).

### 3.4. Multivariate Analysis—Logistic Regression

**Figure 2:**
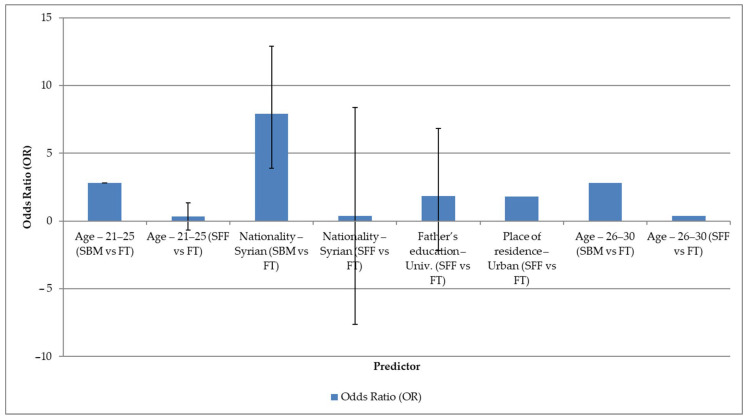
Forest plot of sociodemographic predictors.

In the cross-tabulation analyses, several demographic variables were shown to be correlated with our three attitude cluster groups. After the bivariate analyses, logistic regression was applied with the aim of detrending those factors that remained significant when controlling for other variables (Figure 2).

The results of the model are shown in the forest plot (Figure 2), where the odds ratios and their 95% confidence intervals are presented. In all cases, the reference group was the FT group. The regression analysis reveals that each of the cluster groups has a highly distinguishable demographic profile. This is particularly evident for age, nationality, and the father’s educational level.

For the age groups, respondents aged 21–30 years were significantly more likely to be included in the SBM cluster compared to the FT group (OR ≈ 2.8; 95% CI: 1.07–7.36), while the same age group was rather less likely to be part of the SFF cluster (OR ≈ 0.34–0.36). Gender was found to be a strong predictor: Syrian respondents were much more likely to belong to the SBM attitude cluster compared to the FT group (OR = 7.91; 95% CI: 3.83–16. 31) but were much less likely to belong to the SFF cluster (OR = 0.39; 95% CI: 0.17–0.88).

Respondents whose father had a university degree were more likely to be in the SFF cluster (OR = 1.84; 95% CI: 1.18–2.87). In addition, urban residence also increased the likelihood of being in the SFF cluster compared to the FT cluster (OR = 1.79; 95% CI: 1.08–2.97). No significant association with cluster membership was found for other variables (mother’s educational level, marital status, income status).

## 4. Discussion

It can be noted that IIFAS statements can be grouped by thematic criteria.

1. The participants’ attitudes toward **perceived health and nutritional values** are important. There is no doubt that breastfeeding is the optimal nutrition for the ideal growth, development, and health of infants [1,3,46]. In our study, the responses among the two nationalities are logical and consistent with research, which indicates that breastfed infants generally experience better health outcomes compared to formula-fed infants [1,20,47]. Infants generally digest breast milk more easily than formula because breast milk contains enzymes that facilitate better absorption of nutrients. Additionally, the proteins in breast milk are predominantly whey proteins, which are softer and more easily digestible than the casein-dominant proteins found in many formulas [21,22]. Syrian participants agree more than Hungarians that breast milk is more easily digested than formula, but the percentages align with previous studies [48]. Breast milk indeed contains relatively low amounts of iron [49,50], but it is highly bioavailable, meaning that infants can absorb it efficiently. Thus, students’ choices may differ based on how they interpret the fact of iron bioavailability. There were notable differences in how Hungarian and Syrian students responded to this statement. Most of the Hungarian participants were uncertain. In comparison, Syrian participants were more divided. Thus, these results do not resemble previous studies [48,51]. Potential overfeeding occurs in 37% of fully formula-fed infants [52,53]. However, the high rate of “not sure” responses among the two statements regarding the relationship between feeding methods and overfeeding could reflect the complexity of the topic; still, this finding is consistent with previous research in particular details: agreement and disagreement [54].
2. Another thematic criterion pertains to the **convenience and practical aspects**. According to international organisations, breastfeeding has always been more convenient than formula feeding [1,40], and our study’s results comply with this finding. Although only about half of the participants expressed their agreement with this statement, it aligns with previous studies [51]. Returning to work is one of the significant challenges nursing mothers face and it affects the duration of exclusive breastfeeding [55,56]. Previous studies showed that formula feeding is the best option when the mother plans to return to work [57], which is what we also found in our results.
3. **Bonding and emotional connection** are criteria that can clearly be shown. Research shows that hormones are associated with bonding and emotional closeness between mother and child [58,59]. Skin-to-skin contact is an ideal method for greeting the newborn. It fosters a sense of safety and tranquillity in the infant while initiating the bonding process [15,20,60,61] and positively affecting the mother’s mood [62]. The majority of the participants agreed that breastfeeding increases mother–infant bonding, making their decisions strongly consistent with the results of previous studies [3,6] and with global organisations [15,20]. In addressing emotional connection, fathers have a remarkable role in supporting mothers, and their attitudes usually significantly influence mothers’ decisions to breastfeed [63,64]. Our findings showed that these results are different from the findings of previous studies [51].
4. The last criterion is related to **social and risk perception**. Breastfeeding in public spaces always has conflicting opinions among participants, especially when the “breastfeeding with discretion” phrase is missing or when the location or use of a cover is not determined, and that is why it is noticeable that, in general, there is concord in the responses among the participants, making the approximate percentages consistent with other international research [64,65], and conflict with others who found restrictive attitudes toward exposure to the breast, considering it unacceptable behaviour and to be kept private [66,67]. Consuming alcohol during breastfeeding undoubtedly has effects on both infants and mothers [68,69]. However, current recommendations emphasise waiting a minimum of two hours after consuming alcohol before breastfeeding [70,71]. This fact, which encourages mothers not to stop breastfeeding even if they drink alcohol, considering the recommendations, is unknown to the majority of the participants, making the results consistent with other studies [48].
5. A key result of the research was the appearance of three distinct attitude clusters. These clusters showed clear patterns in the respondents’ attitudes towards different forms of breastfeeding. Syrian respondents were more likely to be in the SBM cluster, i.e., strongly supportive of breastfeeding, while Hungarian respondents were more likely to be in the SFF or FT clusters. Age also emerged as an important predictor, with younger respondents (21–30 years old) more likely to be in the cluster supporting breastfeeding. Although cross-tabulation analyses showed that education level was associated with cluster membership, the regression model results indicated that this association remained insignificant after controlling for other demographic factors. Curiously, the father’s education level was also a statistically significant predictor, particularly for the SFF cluster. These findings emphasise that cultural context and social status strongly shape young adults’ attitudes towards breastfeeding.

## 5. Strengths and Limitations

A key strength of this study is its comparison between Syrian and Hungarian university students, which provides valuable insight into how cultural norms, traditions, and personal experiences influence breastfeeding attitudes among females. Focusing on university students offers an opportunity to examine the attitudes of future mothers, who are likely to play a crucial role in shaping infant feeding practices in their respective societies. Moreover, the use of the Iowa Infant Feeding Attitude Scale (IIFAS), a validated and widely used instrument, enhances the reliability and comparability of the findings by reducing ambiguity and subjectivity in measuring attitudes toward breastfeeding.

However, this study also presents several limitations. The sample size, while adequate for exploratory analysis, may not be representative of the general population in either country, as university students typically have higher education levels and may come from more urban or specific socioeconomic backgrounds. This could influence their perceptions and intentions regarding breastfeeding, potentially limiting the generalisability of the findings. Additionally, the nature of the questionnaire introduces the risk of social desirability bias, particularly concerning culturally sensitive issues such as public breastfeeding, breastfeeding in formal or professional settings, and familial influence on feeding decisions. Lastly, although the IIFAS was translated into Arabic and Hungarian, linguistic and cultural nuances may have affected participants’ understanding of certain items, which could impact the accuracy and consistency of their responses.

## 6. Conclusions

This research points out the role of cultural norms and personal experiences in shaping breastfeeding attitudes among female university students in Syria and Hungary; however, qualitative research in this area would be worthwhile in the future.

The cluster analysis produced distinct groups along the lines of breastfeeding and formula-feeding attitudes. These clusters differed not only in terms of feeding preferences but also in terms of demographic factors (nationality, education, marital status, income differentials, father’s education).

It was found that low-income respondents were more likely to support breastfeeding, probably due to cost effectiveness and perceived benefits. However, higher-income respondents tended to prefer formula feeding or a flexible approach, presumably because of either better access to financial resources or convenience and lifestyle factors.

Differences between clusters by nationality were also clearly visible. Syrians were more in favour of breastfeeding, which may be due to cultural and religious reasons. At the same time, Hungarians preferred a more flexible approach or even the use of a formula, which may indicate that Hungarians tend to have a more modernised, convenience-focused approach.

The findings indicate that attitudes are primarily shaped by perceived health benefits, practical considerations, emotional bonding, and social norms. Cultural context, particularly national and familial background, played a crucial role in clustering students into distinct attitude groups, with Syrian participants showing stronger support for breastfeeding.

The significant role of cultural norms and personal experiences in shaping breastfeeding attitudes is evident in both countries. Syrian students showed stronger agreement with statements reflecting emotional bonding and traditional breastfeeding values, while Hungarian students expressed more uncertainty or disagreement in areas related to digestion, joy of motherhood, and social perceptions. These differences reflect how cultural and experiential factors influence attitudes toward breastfeeding in both contexts.

Understanding these differences is crucial for developing targeted health promotion strategies that encourage informed breastfeeding choices while respecting cultural diversity. Future research should further explore how societal changes, healthcare policies, and exposure to breastfeeding education influence young adults’ perspectives across different regions.

## Figures and Tables

**Table 1:** Summary statistics of study participants (N = 620).

		Frequency	Percentage
Valid	Syrian	317	51.0%
	Hungarian	303	48.7%
	Total	620	99.7%
Missing	System	2	0.3%
Total		622	100.0%

**Table 2:** Socioeconomic characteristics of the Hungarian and Syrian respondents.

Socioeconomic Status	Hungarian	Syrian
Level of education (N = 620)		
BSc	57.1%	79.5%
MSc	37.0%	14.2%
PhD	5.9%	6.3%
Father’s level of education (N = 620)		
University studies	40.6%	45.7%
Non-university studies	59.4%	54.3%
Mother’s level of education (N = 620)		
University studies	45.9%	47.0%
Non-university studies	54.1%	53.0%
Place of residence (N = 619)		
Urban	63.9%	82.3%
Rural	36.1%	17.7%
Marital status (N = 620)		
Married	0.3%	53.6%
Unmarried	99.7%	46.4%
Faculty (N = 620)		
Medical faculty	66.7%	51.4%
Non-medical faculty	33.3%	48.6%
Wealth index (N = 619)		
Less than enough	1.3%	66.8%
Enough	97.4%	32.6%
More than enough	1.3%	0.6%
Age (N = 620)		
21–15	68.3%	57.7%
26–30	31.0%	29.0%
31 and more	0.7%	13.2%

**Table 3:** Responses for Hungarian and Syrian students to IIFAS (N = 620).

The Statement	Disagree ^1^		Neutral		Agree ^2^	
	%	n	%	n	%	n
1. The nutritional benefits of breast milk last only until the baby is weaned from breast milk.	59.4	364	15.8	97	24.8	152
2. Formula-feeding is more convenient than breastfeeding.	67.2	412	17.6	108	15.2	93
3. Breastfeeding increases mother-infant bonding.	2.0	12	2.3	14	95.8	586
4. Breast milk is lacking in iron.	39.8	244	46.3	284	13.9	85
5. Formula-fed babies are more likely to be overfed than are breastfed babies.	20.6	126	38.2	234	41.3	253
6. Formula-feeding is the better choice if a mother plans to work outside the home.	32.9	200	23.5	143	43.6	265
7. Mothers who formula-feed miss one of the great joys of motherhood.	32.4	198	12.6	77	55.0	336
8. Women should not breastfeed in public places such as restaurants.	48.7	298	14.2	87	37.1	227
9. Babies fed breast milk are healthier than babies who are fed formula.	13.7	84	24.2	148	62.1	380
10. Breastfed babies are more likely to be overfed than formula-fed babies.	52.0	317	39.0	138	9.0	55
11. Fathers feel left out if a mother breastfeeds.	66.3	403	21.2	129	12.5	76
12. Breast milk is the ideal food for babies.	2.6	16	9.5	58	87.9	538
13. Breast milk is more easily digested than formula.	6.9	42	33.4	204	59.7	365
14. Formula is as healthy for an infant as breast milk.	46.1	282	31.0	190	22.9	140
15. Breastfeeding is more convenient than formula feeding.	24.5	150	20.3	124	55.2	337
16. Breast milk is less expensive than formula.	6.9	42	7.8	48	85.3	522
17. A mother who occasionally drinks alcohol should not breastfeed her baby.	12.6	77	19.6	120	67.8	415

^1^: Disagree includes ‘strongly disagree’ and ‘disagree’. ^2^: Agree includes ‘strongly agree’ and ‘agree’.

**Table 4:** Distribution of clusters within nationality.

		Cluster Number of Case			Total
		SBM	SFF	FT	
Nationality	Syrian	61.1%	13.3%	25.6%	100.0%
	Hungarian	12.7%	39.1%	48.2%	100.0%
Total		38.5%	25.3%	36.2%	100.0%

**Table 5:** Distribution of clusters within the level of education.

		Cluster Number of Case			Total
		SBM	SFF	FT	
Level of Education	BSc	43.0%	24.7%	32.3%	100.0%
	MSc	28.1%	26.7%	45.2%	100.0%
	PhD	29.7%	27.0%	43.3%	100.0%
Total		38.5%	25.3%	36.2%	100.0%

**Table 6:** Distribution of clusters within the father’s level of education.

		Cluster Number of Case			Total
		SBM	SFF	FT	
Father’s level of education	University studies	35.9%	30.9%	33.2%	100.0%
	Non-university studies	40.5%	21.0%	38.5%	100.0%
Total		38.5%	25.3%	36.2%	100.0%

**Table 7:** Distribution of clusters within marital status.

		Cluster Number of Case			Total
		SBM	SFF	FT	
Marital status	Married	64.1%	12.4%	23.5%	100.0%
	Unmarried	28.2%	30.6%	41.2%	100.0%
Total		38.5%	25.3%	36.2%	100.0%

**Table 8:** Distribution of clusters within income.

		Cluster Number of Case			Total
		SBM	SFF	FT	
Wealth index	Less than enough	63.6%	12.1%	24.3%	100.0%
	Enough	24.3%	32.9%	42.8%	100.0%
	More than enough	16.7%	33.3%	50.0%	100.0%
Total		38.4%	25.4%	36.2%	100.0%

## Data Availability

The raw data supporting the conclusions of this article will be made available by the authors on request. The data are not publicly available due to ethical reason.

## Abbreviations

The following abbreviations are used in this manuscript:

IIFAS	The Iowa Infant Feeding Attitude Scale
SPSS	Statistical Package for the Social Sciences
PASW	Predictive Analysis Software
